# Accessibility and interventions of crisis resolution teams: a multicenter study of team practices and team differences in Norway

**DOI:** 10.1186/s12888-022-03992-2

**Published:** 2022-05-21

**Authors:** Torleif Ruud, Katrine Høyer Holgersen, Nina Hasselberg, Johan Siqveland

**Affiliations:** 1 Division of Mental Health Services, Akershus University Hospital, Lørenskog, Norway; 2 Institute of Clinical Medicine, University of Oslo, Oslo, Norway; 3 Nidelv Community Mental Health Center, Clinic of Mental Health, St. Olavs Hospital, Trondheim, Norway; 4 Department of Psychology, Norwegian University of Science and Technology, Trondheim, Norway; 5 National Center for Suicide Research and Prevention, University of Oslo, Oslo, Norway

**Keywords:** Crisis resolution teams, Team practice, Accessibility, Interventions, Measurement of practice, Team variance

## Abstract

**Background:**

Components of crisis resolution teams’ (CRTs) practices have been defined in recommendations and a fidelity scale, and surveys have reported how team leaders describe CRT practices. However, studies on CRTs have not measured and reported details of the crisis intervention provided to individual service users. The present study aimed to measure how various components of CRT practice were provided to individual service users and differences in practice between CRTs.

**Methods:**

The study was exploratory and part of a prospective multicenter pre-post project on outcome of CRT treatment in Norway. Accessibility and intervention components of 25 CRTs were measured for 959 service users at the first contact after referral and in 3,244 sessions with service users. The data on CRT practice components were analyzed with descriptive statistics and factor analyses, and differences between teams were analyzed using ANOVA and calculating the proportion (intraclass correlation coefficient) of total variance that was due to differences between teams.

**Results:**

One-third of the service users had their first session with the CRT the day of referral and another third the following day. Treatment intensity was mean 1.8 sessions the first week, gradually decreasing over subsequent weeks. Three of ten sessions were conducted in the service user’s home and six of ten in the team’s location. Eight of ten sessions took place during office hours and two of ten in the evening. The CRT provided assessment and psychological interventions to all service users. Family involvement, practical support, and medication were provided to two of ten service users. Between CRTs, significant differences were identified for a substantial proportion of practice components and especially for several aspects of accessibility. Cluster analysis identified two clusters of CRTs with significant differences in accessibility but no significant differences in the use of intervention components.

**Conclusions:**

Measurements of accessibility and interventions provided to individual service users gave a detailed description of CRT practices and differences between teams. Such measurements may be helpful as feedback on clinical practice, for studying and comparing crisis resolution team practices, and in future studies on the association between different outcomes and potential critical elements of crisis interventions.

**Supplementary Information:**

The online version contains supplementary material available at 10.1186/s12888-022-03992-2.

## Introduction

The optimal practice of crisis resolution teams (CRTs) has been defined in national recommendations, professional guidelines, a fidelity scale, and books [1–4]. Key components include rapid response time to referrals, 24/7 operating time, crisis intervention limited to weeks, high intensity of care, home-based care, gatekeeping of acute psychiatric beds, and facilitating early discharge from inpatient care. Estimates of CRT practices in national surveys and assessments of fidelity to the optimal CRT model have revealed numerous differences between teams regarding organization, staffing, target group, hours of operation, and key components listed above, but the provision of key components are seldom measured and analyzed in relation to individual service users [5–12].

Two reviews of studies on outcomes of CRTs have found that information on treatments provided by CRTs is lacking [13, 14]. One review showed that a striking limitation of all the research reviewed was the impoverished description and analysis of moderating variables, including treatments provided by the CRTs and by the services in the control arms [13]. Recommendations for future research from this review were to specify treatment characteristics with greater detail. The second review aimed to identify evidence regarding characteristics of effective CRTs. The authors found some nonconclusive empirical indications for the effect of CRTs’ extended hours of operation and of including a psychiatrist on the team. However, they were unable to draw any confident conclusions about critical components of CRTs from the available quantitative evidence [14]. Thus, in clinical studies of CRT treatment, the intervention studied is the total CRT practice and not specific elements of CRT practices.

One reason for scarce detailed reporting and analyses of CRT treatments is the lack of methods measuring specific CRT treatment components. A review of measures assessing content of mental health services identified 25 measures [15]. None seemed to have been applied in studies on CRT practices. There is a need for measurements and more detailed knowledge of CRT practices and interventions provided to the individual service users.

### Aims

Aims of the study were to measure, describe, and analyze CRTs’ accessibility (response time, intensity/frequency of sessions, place and time for sessions, duration of crisis treatment), CRTs’ interventions (activities in sessions), differences in CRT practices, and whether there were clusters of teams with different patterns of practice.

## Methods

### Design

This exploratory study was part of a multicenter pre-post research project on outcomes of CRT treatment in Norway [16]. The current study reports on the practices of the CRTs from data collected regarding their sessions with the service users.

### Context

All the 19 health trusts with specialized health services for adults in Norway includes a division of mental health services with hospital departments and two to six community mental health centers (CMHCs). Each CMHC has outpatient clinics, mobile teams, and local inpatient wards. Most of the CMHCs has a CRT as one of their mobile teams [16, 17]. The increase of staff in Norwegian CMHCs during the last decade has occurred mainly in CRTs and other types of mobile teams. The CRTs and other CMHC units also collaborate with general practitioners and other primary health and social services in the community. Most health care in Norway are public services financed through taxation and provided by national and municipal service providers, with in-patient services free of charge and fees for outpatient services (including CRTs) limited to a total annual amount of approximately 300 euro.

### Sample and recruitment

A total of 1,040 service users gave written informed consent at the first session to participate in the project, and the 25 CRTs reported data on 3,244 face-to-face sessions with the 959 included service users included in this study. Table 1 shows the sociodemographic characteristics of these service users, as well as referral agency, earlier contact with the CRT, and main psychiatric diagnosis set by the CRT.

**Table 1:** Sociodemographic and clinical characteristics of service users (*N* = 959)

Variables	N	%
***Sex***		
Male	387	40.4
Female	559	58.3
Unknown	13	1.3
***Age group***		
Under 20	50	5.2
20–29	291	30.3
30–39	201	21.0
40–49	163	17.0
50–59	145	15.1
60 and above	96	10.0
Unknown	13	1.4
***Marital status***		
Married/cohabitant/partner	376	39.2
Single	399	41.6
Divorced/separated	137	14.3
Widow/widower	18	1.9
Unknown	29	3.0
***Living with***		
Living alone	310	32.3
With spouse/cohabitant/partner with or without children	349	36.4
With children	78	8.1
With parents	135	14.1
With others/unknown	87	9.1
***Highest completed education***		
Primary school	236	24.6
Secondary school	393	41.0
College/university	247	25.7
Unknown	83	8.7
***Main source of income***		
Income from own work/loan as student	427	44.5
Sick leave	86	9.0
Other out of work compensation or social support	170	17.7
Disability pension	138	14.4
Old age pension	46	4.8
No income/supported by others/unknown	92	9.6
***Referral agency***		
Self-referral by the service user	130	13.6
Family of service user	46	4.8
General practitioner	407	42.5
Physician on call/other primary care in municipality	104	10.9
Specialized mental health services	189	19.6
General hospital clinic/ward	28	2.9
Others/unknown	55	5.7
***Earlier contact with the crisis resolution team***		
Yes	223	23.3
No	714	74.4
Unknown	22	2.3
***Psychiatric diagnoses***		
Psychosis/bipolar	77	8.0
Depression	261	27.1
Anxiety disorder	249	26.0
Substance use disorder	39	4.1
Personality disorder	46	4.8
Other diagnoses	39	4.1
No diagnosis	248	25.9

The 25 participating CRTs represented almost half of Norway’s 56 CRTs. All the 19 health trusts in Norway were invited and encouraged by the project to let their CRTs participate, and the project did not select CRTs for the study or limit any CRT from participating. Two interested CRTs withdrew before study start due to organizational changes or illness absences.

The CRTs were located at CMHCs together with outpatient clinics and, for the most part, with CMHC inpatient wards as well. The populations in the CMHC catchment areas ranged from 40,000 to 130,000. The staffing for a CRT averaged 10.0 full-time equivalents (range 4.0–20.4), and the average full-time equivalents of the major professional groups in a CRT were 5.4 mental health nurses/nurses, 1.5 clinical psychologists, 1.3 psychiatrists/physicians specializing in psychiatry, and 1.0 social workers.

### Measures

Date and hour for receiving the referral and for the first session with the service user were registered on an registration form completed by the team in the first session, and response time was calculated as the time that elapsed from receiving the referral to the first session.

Data on accessibility and interventions during the CRT treatment were registered on a session registration form by a team member immediately following each session regarding date, time of the day, location of session, duration of session, other participants, and 26 possible activities during the sessions (seven assessment activities, 12 treatment activities, seven types of collaboration). The activities were formulated as concrete behaviors to be easy to understand and rate quickly in a consistent way. Each activity was rated on a four-point scale if present (1 = little, 2 = some, 3 = much, 4 = very much) and counted as 0 (not done) if not rated. This was explained in the instructions for completing the form, and as lack of rating 1–4 was recorded as 0, there were no missing ratings of activities.

The session-registration form was designed for completion in 2–3 min by marking only activities that were conducted and a few other aspects. An English translation of the form with a brief instruction is available as online supplementary information (Session registration form for CRTs). Only data on face-to-face sessions was included in the study. Telephone contact with services users were not included in the data analyses because eight CRTs did not register this as they felt it was too demanding. Drafts of both forms were revised after discussions with the crisis resolution teams at a workshop before the start of inclusion and data collection. The team members received brief written guidelines for completion of the forms.

### Data collection

Data were collected between March 2015 and February 2016. As crisis interventions by CRTs are often defined as up to eight weeks, the maximum time included for CRT treatment in the project was defined to eight weeks. For 77 service users (8.0%) the team continued treatment longer than 8 weeks. Immediately following each session, team members were to register characteristics of the session and rate the activities provided. The 111 sessions (3.4%) that were registered after eight weeks treatment were also included in the data analyses.

### Data analysis

We used frequency tables and descriptive statistics to describe the sample of service users and the registered CRT accessibility and activities per session, service user, and team. We analyzed differences between teams using one-way variance analyses (ANOVA), and we calculated the team-level proportion (intraclass correlation coefficient, ICC) of total variance using empty linear mixed models with only intercepts. There are no established guidelines for what should be considered high or low team-level proportion (ICC) of total variance, as this will depend on many factors. However, for these data we defined high ICC as 25.0% and above, medium ICC as 10.0 – 24.9%, and low ICC as below 10.0%.

For activities rated after sessions, we used factor analyses to reduce the 26 session activities to a manageable number of intervention components for use in further analyses. We used principal component analysis with varimax rotation and Kaiser’s criteria of eigen values at 1 or more separately for the seven assessment activities, the 12 treatment activities, and the seven collaboration activities. We assessed the internal consistency of each factor calculating Cronbach’s alpha. According to the guidelines, we interpreted alpha as unacceptable (< 0.70), fair (0.70–0.79), good (0.80–0.89), and excellent (≥ 0.90) [18]. Activities rated 2–4 (some, much, very much) for a session were calculated and reported as provided in the session. Analysis and reporting of provided activities and intervention component were based on the calculated proportion of sessions where the activity or component had been provided, for both service users and teams.

To explore if there were significant differences in practice between CRTs with higher and lower accessibility, we performed a two-step cluster analysis of CRTs based on three accessibility variables at the team level (proportion with first session on referral day, proportion of sessions outside CRT location, proportion of sessions outside office hours). Based on the Schwarz Bayesian Information Criterion (BIC), the cluster quality was assessed as good and slightly higher than if we had included intensity of CRT care (number of sessions per week) as a fourth clustering variable. Differences between the two clusters were tested using t-test and controlled using non-parametric Mann–Whitney U test.

Intraclass correlation coefficients (ICCs) for the portion of variance at the team level were analyzed using STATA version 17. All other analyses were performed using SPSS for Windows version 27.

## Results

### CRT accessibility

Table 2 shows the patterns of measured accessibility to CRT treatment. Three of ten service users had the first session on the day of referral and three of ten on the following day. The first session took place after office hours for three of ten. The sessions were conducted in the CRT’s location for six of ten and in the service user’s home for three of ten. Eight of ten sessions took place during daytime. Difference between CRTs was seen both regarding location and time for the sessions. Six of ten sessions lasted 45–60 min, while most of the others were slightly shorter or longer. In seven of ten sessions outside the CRT’s location and in five of ten at the CRT’s location, two team members participated; there was also differences between teams regarding this. The intensity of CRT treatment, measured as the average number of registered sessions per week, was 1.8 the first week and gradually decreasing thereafter.

**Table 2:** Accessibility: time, duration, location, and intensity of sessions for 959 service users and 3,244 sessions

**Characteristics of access for 959 service users**	N (%)
**Response time: First session on day of referral**	328 (34.2)
First session on the day after referral day	287 (29.9)
First session after 2–3 days	156 (16.3)
First session after 4–7 days	112 (11.7)
First session after more than a week	43 (4.5)
Unknown day of first session	33 (3.4)
**Time of day for first session**	
Daytime (8 am to 4 pm)	714 (74.5)
Evening (5 to 9 pm)	220 (22.9)
Night (10 pm to 7 am)	1 (0.1)
Unknown time of day for first session	24 (2.5)
**Duration of crisis intervention (weeks)**	
1–2 weeks	510 (53.2)
3–4 weeks	202 (21.1)
5–6 weeks	105 (10.9)
7–8 weeks	65 (6.8)
More than 8 weeks	77 (8.0)
**Characteristics of 3,244 registered sessions**	**N (%)**
**Number of sessions per service user**	
One session	214 (22.3)
2–3 sessions	418 (43.6)
4–5 sessions	176 (18.4)
6–10 sessions	124 (12.9)
11 sessions or more	27 (2.8)
**Location of sessions**	
Service user’s home	1064 (32.9)
Local community	130 (4.0)
npatient ward (service user is inpatient)	14 (0.4)
Locations of other services	27 (0.8)
Crisis resolution team’s location	2009 (61.9)
**Proportion of sessions outside CRT location**	1235 (38.1)
**Time of day for start of sessions**	
Daytime (8 am to 4 pm)	2666 (82.2)
Evening (5 to 9 pm)	556 (17.1)
Night (10 pm to 7 am)	4 (0.1)
Unknown time of day for start of session	18 (0.6)
**Proportion of sessions outside office hours**	560 (17.3)
**Duration of sessions (minutes)**	
5–20 min	152 (4.7)
25–40 min	379 (11.7)
45–60 min	1907 (58.8)
65–90 min	644 (19.8)
Unknown duration of session	162 (5.0)
**Proportion of sessions with two members**	1897 (58.5)
**Intensity of crisis intervention (sessions per week)**	**Mean (SD)**
Week 1	1.8 (1.0)
Week 2	1.4 (0.7)
Weeks 3–4	1.2 (0.5)
Weeks 5–6	1.2 (0.6)
Weeks 7–8	1.1 (0.4)

### CRT interventions

Table 3 shows the patterns of session activities and intervention components in the 3,244 sessions with the 959 service users. Factor analysis of assessment activities resulted in one primary factor of assessment and treatment planning, and one factor that we interpreted as the assessment of severe conditions. Factor analysis of the treatment activities resulted in four factors: practical support, psychological interventions, family involvement, and medication management. Factor analysis of collaboration activities resulted in one factor of collaboration with inpatient services and one factor of collaboration with general practitioners and primary care. The internal consistency of the factors calculated as Cronbach’s alpha was unacceptable for five factors and fair for two factors.

**Table 3:** Factor analysis and patterns (descriptive statistics) of activities and intervention components in 3,244 sessions with 959 service users

**Intervention components (factors) and registered session activities within each component**	**Factor loadings**	**Sessions (%) with this**^a^	**Service users (%) with this**^b^
		***N* = 3,244**	***N* = 959**
**Assessment (55.1% of variance explained by factors)**			
**Assessment and treatment planning (Cronbach’s α = .75)**		**2830 (87.2)**	**950 (99.1)**
Examining psychiatric status	.817	(71.8)	(94.2)
Mapping the situation	.772	(60.9)	(94.7)
Assessing suicidal risk	.729	(48.2)	(82.4)
Making a treatment plan	.659	(58.8)	(85.5)
Assessing, setting diagnosis	.419	(18.1)	(41.2)
**Assessment of severe conditions (Cronbach’s α = .37)**		**200 (6.2)**	**168 (17.5)**
Examining physical health	.859	(1.1)	(3.6)
Assessing the risk of violence	.691	(5.3)	(14.8)
**Treatment (54.0% of variance explained by factors)**			
**Practical support (Cronbach’s α = .63)**		**269 (8.3)**	**192 (20.0)**
Providing/arranging help finances/housing	.709	(2.2)	(5.8)
Arranging for practical help	.655	(1.6)	(4.5)
Making a written crisis plan	.615	(1.8)	(5.0)
Assistance regarding work/education	.612	(3.8)	(9.8)
Providing practical help	.571	(1.0)	(2.6)
**Psychological intervention (Cronbach’s α = .54)**		**2870 (88.5)**	**936 (97.6)**
Working through thought/feelings	.781	(63.0)	(81.9)
Clarifying/sorting the situation	.755	(73.5)	(94.5)
Providing information	.537	(49.7)	(78.7)
Psychotherapy	.451	(17.9)	(28.2)
**Family involvement (Cronbach’s α = .78)**		**368 (11.3)**	**253 (26.4)**
Sessions with family/network	.897	(8.5)	(21.0)
Information/guidance to family	.891	(8.2)	(20.6)
**Medication management**	.888	**401 (12.4)**	**205 (21.4)**
**Collaboration (47.8% of variance explained by factors)**			
**Collaboration with hospital/inpatient services (Cronbach’s α = .47)**		**146 (4.5)**	**115 (12.0)**
Contact during inpatient stays	.798	(0.3)	(0.9)
Assist at discharge from inpatient stay	.739	(0.4)	(1.1)
Admission to inpatient mental health ward	.564	(3.3)	(9.4)
Accompany to GP or other services	.532	(1.0)	(2.9)
**Collaboration with GP and primary care (Cronbach’s α = .43)**		**410 (12.6)**	**281 (29.3)**
Cooperate with or give advice to primary care services	.743	(3.2)	(7.9)
Cooperate with or give advice to GP	.695	(3.8)	(9.6)
Referral/transfer to other services	.589	(8.2)	(20.8)

^a^Percentage of sessions where activity was rated 2–5 (some, much, very much) and not 0–1 (nothing, little), and where intervention component was provided. The written instructions (page 2) of the “Session registration form for CRTs” (online supplementary information) stated that lack of rating 1–4 would be recorded as 0 (zero, not done). According to this, there were no missing ratings of activities

^b^Percentage of service users where activity or intervention component was provided to some extent according to the information under footnote “a” above

The component of assessment and treatment planning was provided for all service users and in most sessions, while assessment of severe conditions was provided for a minority of service users and in only a few sessions. Psychological interventions were provided to almost all service users and in most of the sessions. Practical support, family involvement, and medication management were provided to a minority of service users and in a few sessions. Collaboration with other services was also conducted for a minority of service users and in a few sessions and involved more collaboration with GPs and primary care providers than with inpatient services.

### Differences between crisis resolution teams

Table 4 shows descriptive statistics for accessibility and provision of intervention components by the CRTs, as well as differences between teams. ANOVA showed significant differences between CRTs for all nine variables on accessibility and for all eight intervention components, and the variation differed across variables. Calculating intraclass correlation coefficient for the proportion of variance at the team level confirmed significant differences between CRTs. The team level variance was high for accessibility variables measuring the proportion of sessions outside the CRT location and office hours and the proportion of sessions where two team members participated. The team level variance was medium for the number of registered sessions per service user, duration of sessions, treatment intensity measured as number of interventions per week, and duration of crisis intervention measured in weeks. Low variance was found regarding response time, measured as the time elapsing from receiving the referral to the first session, and as the proportion of service users receiving the first session on the day of the referral.

**Table 4:** Difference between 25 crisis resolution teams in accessibility and proportion of sessions with intervention components for 959 service users. Descriptive statistics, ANOVA, and proportion of variance (ICC) on team level

**Measures of accessibility and components of interventions by the crisis resolution teams**	**Mean (SD)**	**ANOVA**		**Variance (ICC)**^a^ **on team level**
		**F**	**p**	
**Accessibility**				
Response time: days from referral to session	1.9 (3.3)	2.706	< .001	9.0%
Proportion with first session on referral day	0.35 (0.48)	3.748	< .001	8.5%
Number of sessions per service user	3.4 (2.7)	9.320	< .001	18.3%
Proportion of sessions outside CRT location	.38 (.49)	58.888	< .001	30.0%
Proportion of sessions outside office hours	.17 (.38)	30.987	< .001	29.9%
Proportion of sessions with two members	.58 (.49)	44.724	< .001	38.7%
Duration of sessions (minutes)	57 (21)	29.619	< .001	21.5%
Duration of crisis interventions (weeks)	3.4 (3.0)	4.589	< .001	14.1%
Intensity of crisis intervention (sessions/week)	1.5 (0.7)	10.840	< .001	19.3%
**Portion of sessions with component**				
Assessment and treatment planning	.87 (.33)	12.421	< .001	16.0%
Assessment of severe conditions	.06 (.24)	2.946	< .001	2.7%
Practical support	.08 (.28)	5.332	< .001	11.6%
Psychological interventions	.88 (.32)	6.178	< .001	19.7%
Family involvement	.11 (.32)	4.297	< .001	8.1%
Medication management	.12 (.33)	16.417	< .001	14.3%
Collaboration with hospital/inpatient services	.05 (.21)	3.429	< .001	0.5%
Collaboration with GP and primary care	.13 (.33)	9.188	< .001	12.7%

^a^Intraclass correlation coefficient (ICC) calculated using empty linear mixed models with only intercepts

The team level variance for the eight intervention components was medium for assessment and treatment planning, practical support, psychological interventions, medication management, and collaboration with primary care. The variance was low for assessment of severe conditions, family involvement, and collaboration with inpatient services, which were also the least-often used components.

### Differences between clusters of crisis resolution teams

Table 5 shows the differences between two clusters of 10 CRTs with higher and 15 CRTs with lower accessibility based on three key accessibility variables as explained in Methods. There were significant differences between the CRT clusters regarding response time (waiting time, proportion of service users with first session on day of referral), access to sessions (proportion of sessions outside the CRT location, proportion of sessions outside office hours), and intensity of CRT care (number of sessions per week). No significant differences were found regarding the number of sessions registered per service user, the duration of sessions, or the duration of CRT treatment. There were no significant differences between CRT clusters for any of the eight intervention components. Non-parametric Mann–Whitney U test gave similar results as the t-test for all accessibility and intervention component variables.

**Table 5:** Accessibility and intervention components for two clusters of crisis resolution teams classified by two-step cluster analysis. Descriptive statistics and t-tests of independent groups

	**Cluster 1 (*N* = 10)**	**Cluster 2 (*N* = 15)**	**Difference**^**a**^
**Variables**	**Mean (SD)**	**Mean (SD)**	**p**
**Accessibility**			
Response time: days from referral to session	1.91 (0.33)	2.41 (0.34)	.001
Proportion with first session on referral day	0.45 (0,16)	0.29 (0.12)	.007
Number of sessions registered/service user	3.68 (1.49)	3.10 (1.25)	.308
Proportion of sessions outside CRT location	0.57 (0.15)	0.16 (0.13)	< .001
Proportion of sessions outside office hours	0.29 (0.17)	0.07 (0.12)	.001
Proportion of sessions with two members	0.72 (0.22)	0.49 (0.28)	.036
Duration of sessions (grouped by duration)	2.99 (0.33)	3.12 (0.26)	.291
Duration of CRT care (weeks)	2.27 (0.72)	2.31 (0.71)	.895
Intensity of CRT care (sessions/week)	1.65 (0.35)	1.31 (0.18)	.004
**Portion of sessions with component**			
Assessment and treatment planning	0.61 (0.07)	0.53 (0.11)	.084
Assessment of severe conditions	0.05 (0.03)	0.05 (0.03)	.984
Practical support	0.03 (0.03)	0.03 (0.03)	.818
Psychological interventions	0.53 (0.08)	0.51 (0.12)	.526
Family involvement	0.12 (0.10)	0.08 (0.05)	.191
Medication management	0.12 (0.12)	0.12 (0.13)	.891
Collaboration with inpatient services	0.02 (0.01)	0.01 (0.01)	.166
Collaboration with GP and primary care	0.06 (0.06)	0.06 (0.04	.888

^**a**^Non-parametric Mann–Whitney U test gave similar results for all variables

## Discussion

In summary, one third of the service users had their first session with the CRT on the day of the referral, and another third had their first session the day after the referral. The crisis intervention lasted for 1–2 weeks for half of the service users and 3–6 weeks for one third. Treatment intensity was mean 1.8 sessions the first week and was gradually reduced over the subsequent weeks. Three of ten sessions took place in the service user’s home and six of ten at the team’s location. Eight of ten sessions were during office hours and two of ten in the evening. The CRT provided assessment and psychological interventions to all service users. Family involvement, practical support, and medication were provided to two of ten service users. There were significant differences between CRTs for all accessibility variables and types of interventions. The proportion of variance at the team level was high for most variables and especially for some aspects of accessibility. Cluster analyses based on three key accessibility variables showed two clusters of CRTs with significant differences in accessibility but no significant differences in use of interventions.

As we have not found any similar study, we are unable to compare the results to those of other studies measuring CRT practices based on data analyses of sessions with service users. However, the main results are discussed below in relation to results in clinical studies and national surveys.

### CRT accessibility

The data collected at the first contact and after each following session provided descriptive statistics on where, when, and how accessibility was for the individual service user and by the CRT. Such results as response time after receiving a referral have often been reported in studies on CRTs’ characteristics and outcomes [9, 19–21]. Estimates of this have also been reported at the group level in national surveys of CRTs [7, 8, 10, 11]. However, the present study also reported specific data on various aspects of accessibility during CRT treatment, and a number of these have often not been reported at the service user level in studies or surveys. They include the number of sessions, the extent of home-based care, the proportion of sessions outside regular office hours, session duration, treatment intensity (sessions per week), and duration of CRT treatment.

### CRT interventions

In studies on CRTs, reporting on interventions has been sparse and less detailed than the reporting on response time and accessibility [6, 9, 12–14, 19–21]. The present study reported information about several aspects of assessments, a range of interventions, and several types of collaborations for each service user. This showed both the proportion of service users who were provided with the intervention and the proportion of sessions where the intervention was provided. The five activities in assessment and treatment planning were closely related and were provided to all service users and during most sessions. Examining physical health and assessing risk of violence were less related and were provided for a minority of service users and in very few sessions, probably for those service users considered to have more severe illnesses or problems.

The provision of the interventions was consistent with the fidelity assessments of the CRTs in the present project, and with an earlier study conducted in Norway [5, 19]. Psychological intervention was provided to almost all service users and in most sessions, which was much higher than in the UK, according to surveys there [8, 10]. Norwegian CRTs have been shown to have longer sessions and a high emphasis on talk therapy, a lower proportion of service users with severe mental illness, and one or more psychologists on every team [19]. Medication was provided to few service users by Norwegian CRTs, which reflects the lower proportion of service users with severe mental illness. Practical support was provided to few service users, and this is consistent with other studies conducted in Norway and to findings in studies of CRTs in the UK [5, 7, 8, 10, 12, 19]. The low family involvement was surprising because there has been enthusiasm in many Norwegian CRTs for an open dialogue approach involving family and their network [22]. The limited collaboration with inpatient services reflected the fact that Norwegian CRTs did not have gatekeeping functions for acute psychiatric beds or facilitated early discharge from inpatient units. There was more collaboration with GPs and primary care, but for most of these service users, the collaboration activity involved the transfer of service users to other services.

The factor analyses grouped the session activities into meaningful intervention components. Still, the internal consistency (Cronbach’s alpha) for five of these components was unacceptable. This means that there were differences between activities within the same component, and that reporting on both session activities and intervention components provided a more comprehensive picture of the CRT interventions. Further development of such measurements might show intervention components with higher internal consistency.

### Differences between crisis resolution teams

There were significant differences between CRTs for all the measurements of CRT practices. The differences were larger for accessibility than for interventions. The accessibility variables are robust and not depending on team members’ assessments. The CRT model is defined based mainly on accessibility, and differences in accessibility show differences in the implementation of the model. The gatekeeping function for acute psychiatric beds is considered an important element in the model, but this is not a variable in Table 3 as no CRT in the present study had a gatekeeping function. The lower implementation of the CRT model in Norway than in the UK was discussed in an article on CRT fidelity of the Norwegian teams [5]. Influenced also by other developments in mental health services delivery, CRTs in Norway have partly aimed for earlier interventions with lower thresholds in crises and a broader target group, and they have partly adapted the CRT model to more rural areas with longer distances for staff or service users to travel. Despite differences between Norwegian and UK CRTs, there are also differences in implementation both in Norway, as shown in the present study, and in a UK study [7].

There were also significant differences between CRTs for all intervention components, although the variances at the team level for these were not as large as for the elements of accessibility. These patterns confirm the results of the fidelity assessments of the same teams regarding use of these interventions [5]. Compared with UK teams, Norwegian teams provided psychological interventions more often and medication less often, while CRTs in both countries are shown to provide little practical support. The differences may be related to differences in the implementation of the CRT model and because the Norwegian teams served a wider service user group and, thereby, providing a broader range of interventions [5].

Similar differences between CRTs for aspects of accessibility (opening hours, response time, intensity of care) and for interventions (psychological interventions, medication management, practical support) was also found in a national survey that included all the CRTs in Norway at that time [12]. However, the current study included more aspects of accessibility and more interventions than the survey had included. Even more important, while the survey collected data on how team leaders described the CRT practices, the current study measured what the CRTs did and thus gave more detailed and substantial information on team practices and on differences between teams.

The two clusters for CRTs differed significantly for most of the accessibility variables but not for any of the intervention components. This is consistent with the CRT model being defined based mainly on accessibility variables, while the content of treatment has been less focused and less specific. It may be that the two clusters of teams had different degrees of focus on implementing the CRT model but a more similar approach to the content of treatment provided in the sessions with the service users. The interventions provided seemed to be much the same as the interventions provided in outpatient clinics in the CMHCs [17].

### The method used to measure components of CRT practices

The first part of the session registration form contained variables regarding location, time, duration, and participants at the session. These variables on accessibility and practical matters were considered robust and not influenced by team members’ assessments, even if occasional errors in completing forms or electronic registering of data cannot be ruled out.

To measure interventions, the team members rated 26 selected activities in each session. Using factor analyses, these session activities were grouped into eight intervention components. Future improvement of measurements of intervention activities might include better definitions of intervention activities to provide higher internal consistency for the intervention components. Another possibility might be to use a Delphi process to reduce the number of intervention activities as conducted for case management activities in a study reducing 38 potential categories to 11 categories [23, 24]. It might also be useful to test what would be easiest for clinicians and would have the best interrater reliability: to rate eight intervention components defined at a more abstract level or to rate 26 more specific intervention activities.

We did not measure and analyze the interrater reliability of the ratings of sessions and do not know whether the results have been influenced by bias or variations in ratings. This was also one of the limitations for several other measures of content of mental health services found in the review of such measures [15]. Retrospectively, we recognize that we could have estimated interrater reliability by asking a sample of CRT members to rate a set of vignettes describing sessions with service users or by having a sample of sessions that were rated independently by the two team members who had participated in the session [25, 26]. Grouping ratings 2–4 as one, as a part of the data analyses of intervention activities provided in sessions, may have reduced the influence of any lower interrater agreement within this range of ratings.

The session registration form combined all three types of measures of the content of mental health services discussed in the review of such measures [15]: event recording measures, time recording measures, and a retrospective questionnaire regarding the content of the mental health services that were provided. The combination of these three types of elements was tailored to cover the specific components of the CRT care. While several other retrospective questionnaires on content of services cover services delivered over a longer period, the intervention activities in the session registration form were completed for the specific session shortly after the session and while it was easy to remember.

One question is if the participating CRTs were representative for all the CRTs in Norway at the time. Except for fewer CRTs from northern Norway, the participating teams were located quite evenly in 15 of the 19 health trusts in urban and rural areas throughout the country. The average staffing of the teams was similar to the average staffing of all CRTs in Norway [12]. Two interested CRTs withdrew before study start due to organizational changes or illness absences. Other nonparticipating CRTs may have been less eager to get feedback or participate in research, felt less able to do data collection in addition to the clinical work, or were already involved in other studies.

We were not able to compare the collected data to data in the electronic patient records in the 15 health trusts. However, from 11 CRTs that were able to provide anonymous data on the portion recruited during three months of inclusion, we know that 28% of their service users (range 7 – 77%) were included in the study. We do not know many who were not included because they chose not to participate or how many who were not included for other reasons (including not being informed about the study due to various reasons). Neither do we know the proportion of sessions of participating service users that the team members collected data on. However, even if less than a third of service users were included and data was not collected on an unknown number of sessions, the data on the substantial number of service users and sessions may still be representative for the CRTs practices.

### Potential use of detailed measurements of accessibility and interventions

More detailed measurements of accessibility aspects and intervention components provided to the individual service users may increase the possibility of analyzing the association between outcomes and various elements of CRT treatment. This might also indicate which components are the most important for different subgroups of service users. Such data collection may also provide more valid and reliable reported data on CRT practices for comparison across sites or countries, and as feedback for further development of CRT practices.

In the review that unsuccessfully aimed to identify characteristics of effective CRTs, the authors discussed how future studies might solve the methodological challenge of exploring the association between outcome and the many components in CRT practices [14]. Stating that it might not be reasonable or feasible to do randomized controlled trials testing the individual effect of each CRT practice component, they suggested that a potential alternative could be to study outcome associated to CRT practice components using multilevel modeling in a multicenter study with many CRTs. The approach in the present study measuring accessibility and intervention components provided to the individual service users could be a part of such studies.

The focus in this study has been on crisis resolution practices and team differences. The analyses we have done of the material available in this study has shown differences between teams both regarding aspects of accessibility and use of intervention components. However, in this study we have not analyzed differences in practices and interventions in relation to individual service users or different outcomes. If future research can document associations between specific outcomes for specific groups of service users and measured CRT components, this could perhaps lead the way to a more “personalized medicine” in mental health crisis interventions.

### Strengths and limitations

Strengths of the study included a CRT sample located evenly in 15 of the 19 health trusts throughout urban and rural areas throughout Norway (except fewer in northern Norway) and data on a substantial number of service users and sessions. Accessibility and intervention components were reported in detail by CRT team members. Limitations include indications that less than one third of potential service users were recruited, an unknown variation regarding how complete the data collection on sessions was, and that telephone calls were not included in the data analyses as eight teams chose to not collect data on these. There was also an unknown bias in how team members rated their activities in the sessions, and we did not measure interrater reliability. Other choices regarding data analyses and cut-offs for ratings to define the provision of an intervention might have shown different results. The measured results of accessibility and interventions were not analyzed in relation to CRT fidelity assessment and ratings. The generalizability to CRTs in other countries may be limited due to possible differences in the composition of service users and staffing and treatment cultures.

## Conclusions

Measurements of several aspects of accessibility and interventions provided a detailed description of patterns of crisis resolution team practices and of differences between teams. Such measurements may be helpful as feedback to clinicians, in studying and comparing crisis resolution team practices, in future research on the association between outcomes and various components of CRT practices, and for policy makers and leaders developing the mental health services further.

## Supplementary Information

**Additional file 1.** 

## Data Availability

The datasets generated and/or analyzed during the current study are not publicly available due to restrictions in the informed consent signed by the participants but are available from the corresponding author on reasonable request.

## Abbreviations

CRT: Crisis resolution team
CMHC: Community mental health center
ICC: Intraclass correlation coefficient
