# Supplementary material for: Accessibility and interventions of crisis resolution teams: a multicenter study of team practices and team differences in Norway
Source: BMC Psychiatry. 2022 May 21;22:350. doi: 10.1186/s12888-022-03992-2 (PMC9123690; doi:10.1186/s12888-022-03992-2)
Supplement: Supplementary file 1 — Additional file 1. [file 12888_2022_3992_MOESM1_ESM.pdf]

| Session registration form for crisis resolution teams | Code for team | |
|---|---|---|

| | |
|---|---|
| Service user code number | |
| Date for session (dd.mm) | |
| Completed by | |
| Second from team | |
| Third from team | |

**Location for session (mark)**

- ☐ 1 Home of service user
- ☐ 2 Elsewhere in local community
- ☐ 3 Location of the team
- ☐ 4 At inpatient ward (code: ____ see list→)
- ☐ 5 At other service (code: ____ ser list→)
- ☐ 6 Telephone/video meeting

Mark here if service user did **not** participate: ☐

**Others who participated (mark all)**

- ☐ 1 Spouse/cohabitor/partner
- ☐ 2 Others in family (parents/children/siblings)
- ☐ 3 Friends/others in informal network
- ☐ 4 General practitioner (GP)
- ☐ 5 Primary care mental/substance use service
- ☐ 6 Social welfare or employment service
- ☐ 7 CMHC outpatient clinic or other team
- ☐ 8 CMHC inpatient ward or crisis beds
- ☐ 9 Hospital inpatient unit mental health
- ☐ 10 Substance use outpatient clinic/team
- ☐ 11 Outpatient clinic general health
- ☐ 12 Inpatient unit general hospital
- ☐ 13 Employer/staff from educational institution
- ☐ 14 Others:
- ☐ 15 Others:

Mark closest hour for start of session

| 01 | 02 | 03 | 04 | 05 | 06 | 07 | 08 | 09 | 10 | 11 | 12 | 13 | 14 | 15 | 16 | 17 | 18 | 19 | 20 | 21 | 22 | 23 | 24 |
|---|---|---|---|---|---|---|---|---|---|---|---|---|---|---|---|---|---|---|---|---|---|---|---|
| | | | | | | | | | | | | | | | | | | | | | | | |

Mark duration of session in minutes (Write here if more than 120 minutes: _____ )

| 5 | 10 | 15 | 20 | 25 | 30 | 35 | 40 | 45 | 50 | 55 | 60 | 65 | 70 | 75 | 80 | 85 | 90 | 95 | 100 | 105 | 110 | 115 | 120 |
|---|---|---|---|---|---|---|---|---|---|---|---|---|---|---|---|---|---|---|---|---|---|---|---|
| | | | | | | | | | | | | | | | | | | | | | | | |

| | | Focus and activities in session (circle code for each relevant items) | Little | Some | Much | Very much |
|---|---|---|---|---|---|---|
| Assessment | 1 | Mapping situation and network | 1 | 2 | 3 | 4 |
| | 2 | Assessing psychiatric status, assess the severity of the condition | 1 | 2 | 3 | 4 |
| | 3 | Diagnostic assessment | 1 | 2 | 3 | 4 |
| | 4 | Assessing risk of suicide | 1 | 2 | 3 | 4 |
| | 5 | Assessing risk of violence | 1 | 2 | 3 | 4 |
| | 6 | Examining physical health | 1 | 2 | 3 | 4 |
| | 7 | Making a treatment plan | 1 | 2 | 3 | 4 |
| Treatment | 8 | Providing information about illness and/or possible help | 1 | 2 | 3 | 4 |
| | 9 | Clarifying/sorting the current situation | 1 | 2 | 3 | 4 |
| | 10 | Exploring/working with thoughts and feelings | 1 | 2 | 3 | 4 |
| | 11 | Psychotherapy | 1 | 2 | 3 | 4 |
| | 12 | Family/network conversation with service user and next of kins | 1 | 2 | 3 | 4 |
| | 13 | Information/guidance for family/next of kins | 1 | 2 | 3 | 4 |
| | 14 | Prescribing/providing medication | 1 | 2 | 3 | 4 |
| | 15 | Providing practical help (shopping/cooking/housework/cleaning) | 1 | 2 | 3 | 4 |
| | 16 | Arrange practical help from community services | 1 | 2 | 3 | 4 |
| | 17 | Providing/arranging for help with finances/housing/personal rights | 1 | 2 | 3 | 4 |
| | 18 | Discussing or providing assistance regarding work or education | 1 | 2 | 3 | 4 |
| | 19 | Preparing a written plan for relapse prevention | 1 | 2 | 3 | 4 |
| Collaboration | 20 | Following service user to the GP, dentist, or other service | 1 | 2 | 3 | 4 |
| | 21 | Collaborating with and/or providing advice to GP | 1 | 2 | 3 | 4 |
| | 22 | Collaborating with and/or providing advice to community services | 1 | 2 | 3 | 4 |
| | 23 | Preparing/implementing admission to inpatient mental health unit | 1 | 2 | 3 | 4 |
| | 24 | Staying in touch during an inpatient stay in mental health unit | 1 | 2 | 3 | 4 |
| | 25 | Planning/assisting with discharge from inpatient mental health unit | 1 | 2 | 3 | 4 |
| | 26 | Planning/implementing referral/transfer to another health service | 1 | 2 | 3 | 4 |

Additional material to:
Ruud T, Holgersen KH, Hasselberg N, Siqveland J. Accessibility and interventions of crisis resolution teams: A multicenter study of team practices and team differences in Norway. BMC Psychiatry, 2022

**Instructions for completing Session registration form for crisis resolution teams**

The form for the sessions (and meetings/telephone calls) will provide information about the type of services provided by the team to the service user in this session. It is designed so that it is quick to fill in by usually just ticking boxes or putting a circle around numbers.

The form must be competed after each session (or meeting or telephone call) which is more than just giving or receiving a message. This applies to all session with the service user from admission to discharge. The form must also be completed for meetings or telephone conversations that are part of the treatment even if the service user is not present.

Completion of date, time and duration provides information on how the sessions are distributed on weekdays and time of the day, and on duration of sessions and frequency of sessions during the treatment. Information on location for the session will show how the ambulant work of the teams. Information about others who participate will show collaboration with others.

There are fields to register up to three team members who participated in the session. In these fields, one must write the unique code of the individual team member that the local coordinator has clarified with the team member. These data will be used to analyze the tasks and roles of different professional groups in crisis resolution teams. There will be no data analysis on any individual team member.

To complete registering of the content of the focus and activities in the session (lower half of the form), you only circle around one number for each activity that was a part of the specific session. Where there is no circle around a number, we will count it as 0 (not focus/activity in this session). These ratings will describe the team's practice and the treatment provided to the service user.

Each completed form for a session is delivered to the local coordinator. Before delivery, please check that all parts of the form have been completed. The coordinator will also review if there is any missing information, and in that case requests additional information.
